# Comprehensive Analysis of Single Nucleotide Polymorphisms in Human MicroRNAs

**DOI:** 10.1371/journal.pone.0078028

**Published:** 2013-11-05

**Authors:** Miao Han, Yun Zheng

**Affiliations:** 1 State Key Laboratory of Genetic Engineering and Institute of Developmental Biology and Molecular Medicine, School of Life Sciences, Fudan University, Shanghai, China; 2 Faculty of Life Science and Technology, Kunming University of Science and Technology, Kunming, Yunnan, China; Institute of Molecular Medicine, Taiwan

## Abstract

MicroRNAs (miRNAs) are endogenous small non-coding RNAs that repress their targets at post transcriptional level. Single Nucleotide Polymorphisms (SNPs) in miRNAs can lead to severe defects to the functions of miRNAs and might result in diseases. Although several studies have tried to identify the SNPs in human miRNA genes or only in the mature miRNAs, there are only limited endeavors to explain the distribution of SNPs in these important genes. After a genome-wide scan for SNPs in human miRNAs, we totally identified 1899 SNPs in 961 out of the 1527 reported miRNA precursors of human, which is the most complete list of SNPs in human miRNAs to date. More importantly, to explain the distributions of SNPs existed in human miRNAs, we comprehensively and systematically analyzed the identified SNPs in miRNAs from several aspects. Our results suggest that conservation, genomic context, secondary structure, and functional importance of human miRNAs affect the accumulations of SNPs in these genes. Our results also show that the number of SNPs with significantly different frequencies among various populations in the HapMap and 1000 Genome Project data are consistent with the geographical distributions of these populations. These analyses provide a better insight of SNPs in human miRNAs and the spreading of the SNPs in miRNAs in different populations.

## Introduction

MicroRNAs (miRNAs) are endogenous small non-coding RNAs that control the translation and stability of mRNAs at transcriptional level [1]. MiRNAs are initially transcribed in the nucleus as long primary transcripts (pri-miRNAs) and further processed by the RNase III Drosha to miRNA precursors with typical hairpin structure [2]. Then, the pre-miRNAs are exported to the cytoplasm by exportin 5 [3] and processed into about 21 nt miRNA duplexes by RNase Dicer [4]. One strand from the miRNA duplex is preferentially selected and loaded onto the RNA-Induced Silencing Complex (RISC) to produce a functional mature miRNA [5]. Mature miRNA then recognizes its target mRNAs mainly by base-pairing between the second to eighth nucleotides (seed region) from its 5′ end and the complementary nucleotides on the 3′ untranslated region (3′ UTR) of target mRNAs [6]. It is well known that miRNAs are involved in various biological processes and diseases [7], [8], [9]. Furthermore, it is estimated that the expression of about one-third of all protein-coding genes are regulated by miRNAs [10].

Single Nucleotide Polymorphism (SNP) is a common type of DNA sequence variation. SNPs in miRNA genes can affect the function of them by modulating the transcription of the primary transcripts, processing of pri-miRNAs and pre-miRNAs, maturation, or miRNA-mRNA interactions [11], [12]. Consequently, SNPs in some miRNAs may lead to various diseases, such as chronic lymphocytic leukemia [13], papillary thyroid carcinoma [14], progressive hearing loss [15], and breast cancer [16], [17].

With the rapid development of sequencing technologies, a wealth of information on human genome variations has been dug out [18]. Over the past years, genome-wide association studies (GWAS) have revealed a large number of genetic variants related to diseases and/or traits and the functional roles of these variants have been studied mostly in the context of protein-coding genes [19]. However, at least one third of the identified variants are within the non-coding intervals [19], which makes it an urgent task to characterize SNPs in non-coding RNAs, such as miRNAs. Till now, several studies have focused on the identification of SNPs in human miRNAs [18], [20], [21], [22], [23], [24]. However, there are some limitations in these studies. First, some papers only listed the frequencies of SNPs in miRNA genes based on the HapMap and 1000 Genomes Project data without analyzing them deeply [18], [23]. Second, even more importantly, there are almost no systematical endeavors dedicated to the explanation of the distribution of SNPs in human miRNAs except [22]. Gong *et al.*, [22] compared the distribution of SNPs between conserved and non-conserved miRNAs, clustered and individual miRNAs, and also between miRNAs in *intragenic* and *intergenic* regions [22]. However, this study ignored the fact that the numbers of SNPs in miRNA genes and the lengths of pre-miRNAs are different.

To fill the gap between the importance of the miRNAs and the lack of knowledge of why the miRNAs have accumulated SNPs in their patterns, we here conducted a global analysis of SNPs in miRNA genes, and identified 1899 SNPs in 961 out of the 1527 pre-miRNAs of human genome. To the best of our knowledge, this represents the most complete list of SNPs in human miRNAs to the date. Even more importantly, we explored the underlying reasons for the distribution of SNPs in miRNA genes from five aspects. First, because conserved genes generally have important functions, we categorized the degree of conservation for one miRNA family based on the number of species in which it appeared and compared the SNP density between miRNA groups with different degree of conservation. Second, accumulating evidences reported that clustered miRNAs (miRNAs locate near each other) are often, though not always, coexpressed with neighboring miRNAs and host genes [25], suggesting that the clustered miRNAs are essential in regulating complex cell signaling networks. Therefore, we compared the average SNP densities between clustered and individual miRNAs. Third, fragile sites are specific loci that appear as constrictions, gaps, or breaks on chromosomes from cells exposed to partial inhibition of DNA replication [26]. It has been reported that human miRNA genes are frequently located at fragile sites [27]. We checked the genomic localization of miRNAs in fragile sites, and then investigated the enrichment of the miRNAs with multiple SNPs in fragile sites. Fourth, our analysis showed that different substitutions of the SNPs in miRNAs have different frequencies which was attributed to their contributions to the stabilities of the secondary structures of pre-miRNAs. Fifth, we also investigated the relationships between the SNP densities of miRNAs and the number of diseases that they were associated with, and the number of QTLs that they were overlapped with, respectively.

## Materials and Methods

### Data Sets

The SNP information (including chromosomal locations and alleles information) was downloaded from the NCBI dbSNP database (build 137 for human) [28]. It should be noticed that the SNPs in the dbSNP database are not only necessarily SNPs but also include the indels, microsatellites. The genomic locations and sequences information of pre-miRNAs and mature miRNAs were obtained from the miRBase database (release 18.0, November 2011) [29]. There are 1527 human pre-miRNAs (of which 1523 pre-miRNAs have location information) and 1921 human mature miRNAs (of which 1919 mature miRNAs have location information) in the miRBase. The genomic coordinates of protein-coding genes and the human genome sequence were downloaded from the UCSC annotation database (Genome Reference Consortium Human Build 37, GRCh37) [30]. The miRNA family information was obtained by counting the number of species with a miRNA family from the miRBase. The cytoband information of human fragile sites was manually collected from the NCBI Gene database (http://www.ncbi.nlm.nih.gov/gene). The detailed locations of cytobands were downloaded from the UCSC annotation database. The alleles of chimpanzee are regarded as ancestral alleles and ancestral alleles for SNPs in all human miRNAs are obtained from from the UCSC database (ftp://hgdownload.cse.ucsc.edu/goldenPath/hg19/database/snp137OrthoPt3Pa2Rm2.txt.gz ). 1545 SNPs in human miRNAs have ancestral alleles information. The relationships between miRNAs and diseases were downloaded from Human MiRNA & Disease Database (HMDD), which stored manually retrieved associations of miRNAs and diseases from literatures [31]. Human QTLs were retrieved from the Rat Genome Database (RGD) (http://rgd.mcw.edu/) [32]. The HapMap data were downloaded from the HapMap ftp site (ftp://ftp.ncbi.nih.gov/hapmap/frequencies/2010-08_phaseIIIII). The data of the 1000 Human Genome Project (release 2012 February) were downloaded from its ftp site (ftp://ftp.ncbi.nih.gov/1000genomes/ftp/release/20110521).

### Definition of SNP Density

The SNP density, as defined previously [22], was defined as below
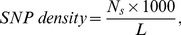
(1)where 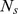 was the number of SNPs in the sequence, 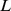 was the length of the sequence (basepair).

### Definition of MiRNA Clusters

The distances of miRNA genes were calculated on the same chromosome and strand based on the reported coordinates in the miRBase. If the distance between two neighboring miRNAs in the same chromosome and strand was smaller than 10 kb, then they were grouped into a cluster. This definition about miRNA clusters is based on the study of miRNA genomic distribution [33]. It has been revealed that the distances between miRNA pairs located consecutively in genome are following a biomodal distribution [33]. The valley between the two peaks is located at around 10 kb, suggesting that 10 kb may be the reasonable cutoff to define miRNA clusters [34].

### Definition the Degree of Conservation for MiRNAs

The miRBase has collected 1315 miRNA families from 153 species. We used the number of species in which a miRNA family appeared to evaluate the conservation of this family. Specifically, if one family appeared in at least 10 species, then it was considered as a highly conserved family. If one family was involved in more than one species but less than 10 species, then it was defined as a lowly conserved family. If a miRNA family only appeared in one species based on the present knowledge, then it was treated as non-conserved.

### Define Influence of SNPs to the Secondary Structures of Pre-miRNAs

RNAfold was used to predict the secondary structures of pre-miRNAs [35]. The minimum free energy (MFE) calculated from RNAfold was used to measure the stability of the secondary structures of pre-miRNAs. Specifically, 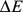 defined in Equation 2 was used to measure the influence of different substitutions to the secondary structures of pre-miRNAs.
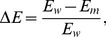
(2)where 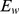 was the minimum free energy of pre-miRNA with ancestral allele, 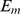 was the minimum free energy of the pre-miRNA with mutated allele. It should be noted that we regarded the pre-miRNA sequences download from the miRBase as the ancestral, the alleles in them as ancestral alleles. Therefore, the ancestral alleles were not necessary the major alleles here.

### Statistical Analyses

The enrichment analysis of miRNAs with at least two SNPs in all fragile sites was evaluated based on the 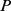-value of the hypergeometric test as follows:
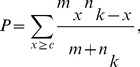
(3)where 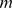 was the total number of miRNAs in all fragile sites, 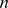 was the total number of miRNAs out of fragile sites, 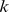 was the total number of miRNAs with at least 2 SNPs, and 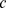 was the number of miRNAs with at least 2 SNPs that located in fragile sites. The enrichment analyses of miRNAs with at least two SNPs in common and rare fragile sites were also preformed in the similar way, respectively.

To compare whether the medians of SNP densities of pre-miRNAs, mature miRNAs and seed regions were significantly different, we performed the Mann-Whitney test for the 961 pre-miRNAs with at least one SNP. The Mann-Whitney test was also used to compare the medians of SNP densities between the 1523 pre-miRNAs and the human genome. To assess the average SNP densities of miRNA families with different degree of conservation, the 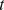-test was used. To assess the average SNP densities of clustered miRNAs, individual miRNAs and the flanking regions between clustered miRNAs, the 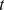-test was also used. The Mann-Whitney test was also used to compare the medians of SNP densities between the miRNAs in and out of all fragile sites, common fragile sites and rare fragile sites, respectively. The 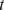-test was also used to compare the average SNP densities between the miRNAs associated with at least one disease or not in the HMDD database.

Spearman’s rank correlation test was used to examine the significance of correlations between: (1) the number of species in which one family appeared and the average SNP density of all the pre-miRNAs, mature miRNAs and seed regions respectively; (2) the number of SNPs and the number of associated diseases of miRNAs; and (3) the number of QTLs overlapped with miRNAs and the number of SNPs in miRNAs.

The 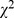-test was used to evaluate the SNPs with significantly different frequencies among various populations and the 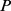-value was calculated by a monte carlo simulation with 10000 replicates [36]. False Discovery Rate (FDR) was used to control the false positive rate of multiple tests [37]. The 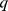-value of FDR less than 0.01 was considered to be statistically significant.

## Results and Discussion

### Global Analysis of SNPs in Human MiRNA Genes

**Figure 1:**
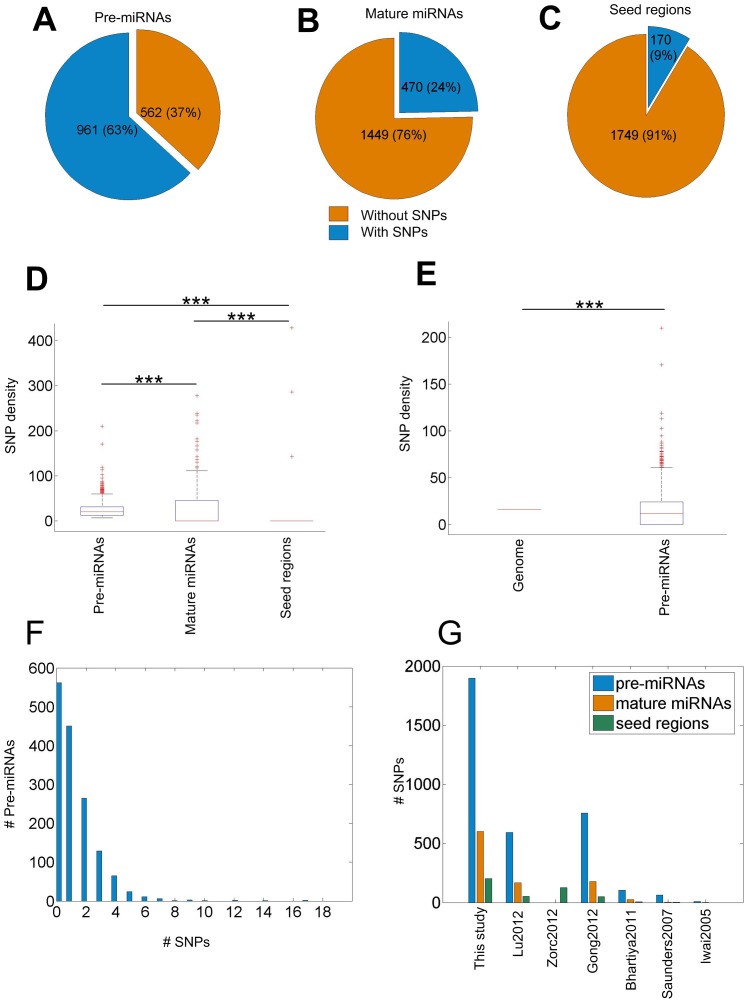
Global analysis of SNPs in human miRNA genes. The proportions of pre-miRNAs, mature miRNAs and seed regions with and without SNPs are shown in part A to C, respectively. Part D shows the distributions of SNP densities in pre-miRNAs, mature miRNAs and seed regions. One tailed Mann-Whitney test of two samples was used to compare the medians of SNP densities among pre-miRNAs, mature miRNAs and seed regions. Only the pre-miRNAs with at least one SNP were used to calculate values in part D and E. Part E shows the distribution of SNP densities of all the pre-miRNAs and the SNP density of the human genome. Part F shows the distribution of the number of SNPs for all the pre-miRNAs. Part G shows the number of SNPs found in pre-miRNAs, mature miRNAs and seed regions in the current study and the other 6 studies [18], [20], [21], [22], [23], [24]. In part D and E, *, ** and *** means 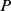-values smaller than 0.05, 0.01 and 0.001, respectively.

Some studies have identified a large number of SNPs in human miRNAs [18], [20], [21], [22], [23], [24]. However, these studies only listed the frequencies of SNPs in miRNA genes based on the HapMap and 1000 Genomes Project data without analyzing them deeply and no systematical endeavors were dedicated to the explanation of the distribution of SNPs in human miRNAs except [22]. However, different numbers of SNPs in miRNAs were not taken into consideration in [22]. After mapping the 50,939,223 SNPs in the dbSNP database to 1527 human miRNA genes, we identified 1899 SNPs (678 validated) in 961 pre-miRNAs, which account for 63% of the 1523 reported pre-miRNAs (see Figure 1A and Table S1). Among them, 601 SNPs (182 validated) are located in 470 mature miRNA sequences, which account for 24% of all 1919 reported mature miRNAs (see Figure 1B and Table S2). Finally, 203 SNPs (68 validated) are located in the seed regions (2 to 8 nt from 5′ end) of 170 mature miRNAs, which only represent 9% of 1919 reported seed regions (see Figure 1C and Table S3). The remaining 562 pre-miRNAs have no SNPs, presumably due to the essential regulatory roles of them. For example, all members of the let-7 family, which play critical roles in many biological processes [38], [39], [40], [41], have no SNPs in their precursors.

Next, we focused on the 961 pre-miRNAs with at least one SNP, and then compared the SNP densities of their pre-miRNAs, mature miRNAs, and seed regions. Figure 1D shows that the median of SNP densities of pre-miRNAs is significantly higher than those of mature miRNAs and seed regions (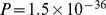 and 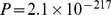, respectively, one tailed Mann-Whitney test). The median of SNP densities of mature miRNAs is also significantly higher than that of seed regions (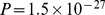, one tailed Mann-Whitney test). These observations are consistent with the function mechanism of miRNAs. As shown in Figures 1A–D, because the mature miRNAs and seed regions are the functional segments of the whole molecules thus they tolerate less SNPs than other regions of pre-miRNAs. From Figure 1E, we can find that the median of SNP densities of all the 1523 pre-miRNAs is significantly lower than the SNP density of the human genome (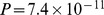, Mann-Whitney test), suggesting the functional importance of the regions of miRNAs. The distribution of the number of SNPs in pre-miRNAs is shown in Figure 1F, where it can be seen that most miRNAs (67%, 1013/1523) have less than 2 SNPs. These results suggest that most miRNAs have important functions and allow no or only 1 SNP. There is one gene pair called miR-4477a and miR-4477b, which is located in the complementary strands at the same location of chromosome 9, have the largest number of SNPs (17) in their pre-miRNA regions, respectively.

We also compared the identified SNPs in miRNAs with those reported in literature as shown in Figure 1G. Iwai *et al.* (2005) revealed 10 SNPs in the 173 human pre-miRNAs without any in the functional seed regions, shown in bars marked as Iwai2005 in Figure 1G [21]. Similarly, Saunders *et al.* (2007) only identified 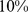 (65/474) pre-miRNAs with SNPs, and found that 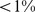 (3/474) of miRNAs have SNPs in functional seed regions, shown in bars marked as Saunders2007 in Figure 1G, presumably due to limited data at the time [20]. Bhartiya *et al.* (2011) identified 106 SNPs mapping to 85 miRNAs based on the miRBase (release 13.0) and dbSNP (build 130), shown in bars marked as Bhartiya2011 in Figure 1G [18]. Gong *et al.* (2012) also identified 757 SNPs in 440 pre-miRNAs based on the miRBase (release 16.0) and dbSNP (build 132), shown in bars marked as Gong2012 in Figure 1G [22]. Zorc *et al.* (2012) identified 149 SNPs in the seed regions of miRNAs in six vertebrates species, and 128 of these 149 SNPs belong to human, shown in bars marked as Zorc2012 in Figure 1G [24]. Lu *et al.* (2012) identified 594 SNPs (169 in mature miRNAs and 54 in seed regions) located inside miRNA precursors (including indels) from 36.8 million SNPs and 3.8 million indels in the 1000 Genome Project, shown in bars marked as Lu2012 in Figure 1G [23]. As shown in Figure 1G, the number of SNPs in pre-miRNAs, mature miRNAs and seed regions identified in the current study are larger than all previous studies, which provides a more comprehensive repository for the study of SNPs in human miRNAs. Even more importantly, existing studies lacks systematical analyses to explain why human miRNA genes display their distribution of SNPs except some analyses without considering the different numbers of SNPs in different miRNAs in [22]. Therefore, we analyzed the miRNA SNPs from several aspects in the following sections as an endeavor to elucidate the patterns of SNP distribution in human miRNAs.

### Conserved MiRNAs Tend to Have Lower SNP Densities

A recently study noticed that conserved miRNAs tend to have fewer SNPs [22]. This study simply grouped miRNAs into with or without SNPs and only considered the conservation of miRNAs in primates or mammals. But different miRNAs have shown big differences in the number of SNPs in them, as shown in Figure 1F, and the lengths of pre-miRNAs are also different (Figure S1). To overcome these limitations, we carefully examined the number of species in which a miRNA family appears and also used the SNP density (as defined in Equation 1) to examine the relation between conservation and the number of SNPs in miRNAs.

**Figure 2:**
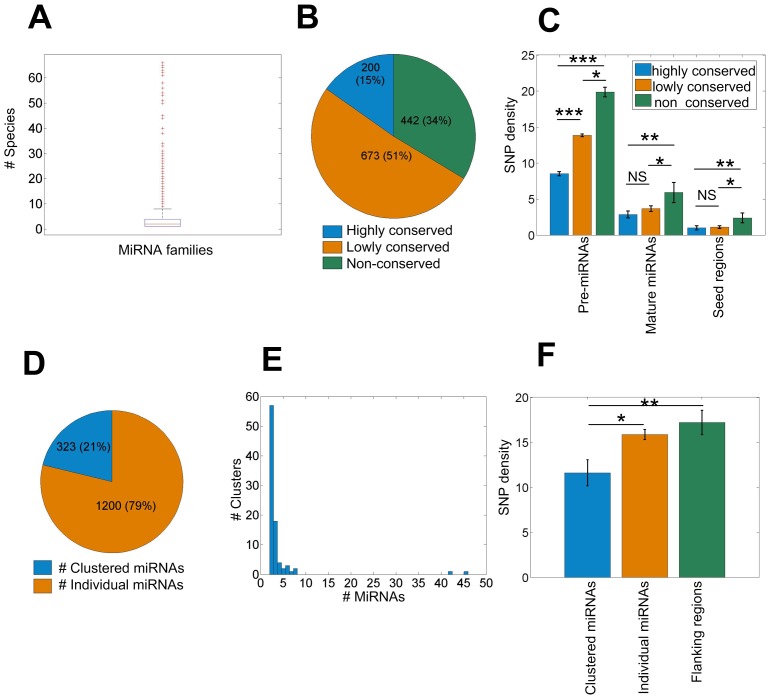
The SNP densities of different regions of miRNAs and different categories of miRNAs. Part A shows the distribution of the number of species in which each miRNA family appears. Part B shows the proportions of human miRNAs classified as highly conserved, lowly conserved and non-conserved miRNA families, respectively. Part C shows the comparing of SNP densities of human miRNAs among highly conserved, lowly conserved and non-conserved miRNA families in the pre-miRNAs, mature miRNAs and seed regions, respectively. Part D shows the proportion of clustered miRNAs. Part E shows the distribution of the numbers of clustered miRNAs in each miRNA cluster. Part F shows the comparisons of SNP densities between clustered miNRAs and individual miRNAs, and also between clustered miRNAs and the flanking regions of clustered miRNAs. Two sample one tailed 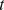-test was used to compare the difference of SNP densities above. In part C, F, *, ** and *** means 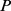-values smaller than 0.05, 0.01 and 0.001, respectively, and error bars indicate the standard errors of the means (SEM).

It was shown in Figure 2A that around 3 quarters of all miRNA families appeared in less than 10 species. Therefore, we classified all miRNA families into highly, lowly and non-conserved if a miRNA family appears in more than or equal to 10, 2 to 9 and 1 species, respectively. Based on this criterion, 200, 573 and 442 human miRNAs were classified to highly conserved, lowly conserved and non-conserved miRNA families, respectively (see Figure 2B). The SNP densities of pre-miRNAs, mature miRNAs and seed regions of the classified miRNA families are shown in Figure 2C and listed in Table S4 to S6. Figure 2C shows that both the highly and lowly conserved families have significantly lower average SNP densities than that of non-conserved families in pre-miRNA regions (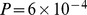 and 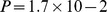, respectively, one tailed 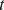-test). The average SNP density of highly conserved families is significantly lower than that of lowly conserved families (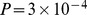, one tailed 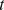-test). We also find a significant negative correlation (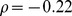, 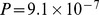, Spearman’s rank correlation test) between the number of species in which one family appeared and the average SNP density of all the pre-miRNAs included in this family. These results suggest that the more conservative one miRNA family is, the less SNPs it can tolerate in pre-miRNA region, which is consistent with the more important functions of the conserved miRNAs. Figure 2C also demonstrates that both the highly and lowly conserved families have significantly lower average SNP densities than that of non-conserved families in mature miRNAs (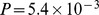 and 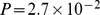, respectively, one tailed 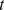-test) and seed regions (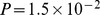 and 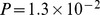 respectively, one tailed 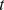-test). However, there is no significantly difference between the average SNP densities of highly and lowly conserved families in mature miRNAs (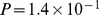,one tailed 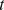-test) and seed regions (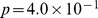,one tailed 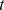-test). There is also no significant negative correlations (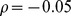, 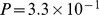, and 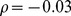, 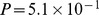, respectively, Spearman’s rank correlation test) between the number of species in which one family appeared and the average SNP densities of all mature miRNAs and seed regions included in a miRNA family, respectively.

### Clustered MiRNAs Tend to Have Lower SNP Densities

One previous paper pointed out that clustered miRNAs tend to have fewer SNPs [22]. However, they only compared the number of miRNAs with or without SNPs but did not consider the fact that different miRNAs may have different numbers of SNPs and different lengths. As demonstrated in Figure 1F, about 33% pre-miRNAs have more than one SNP, which can not be neglected in analysis. Using a maximal distance of 10 kb as a criterion of clustering miRNAs, we identified 89 miRNA clusters containing 323 miRNAs, accounting for 21% of all the analyzed 1523 human miRNAs from miRBase (release 18.0) (see Figure 2D and Table S7). Furthermore, the number of clustered miRNAs in each cluster ranges from 2 to 46 (see Figure 2E). Figure 2F shows that the average SNP density of clustered miRNAs is significantly lower than that of individual miRNAs (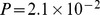, one tailed 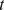-test). This may reflect the critical biological functions regulated by clustered miRNAs to some degree [42], [43], [44]. In the same way, we found that the average SNP density of clustered miRNAs is also significantly lower than that of the flanking regions between them (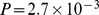, one tailed 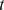-test). When employing 20 kb as the threshold to cluster miRNAs, we also find similar significant results to those using 10 kb (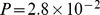 and 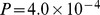, one tailed 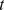-test, respectively).

### MiRNAs With Multiple SNPs are Enriched in Fragile Sites

As shown previously in Figure 1F, 510 pre-miRNAs have more than 1 SNP. We hypothesized that these miRNAs accumulated more than 1 SNP probably partially due to their special genomic contexts. One previous study noticed that miRNAs tend to locate in fragile sites [27]. A recent study focused on miRNAs with seed region polymorphisms and found that 3 miRNAs overlapped with 2 fragile sites [24]. However, no endeavors were given to systematically analysis of SNPs of miRNAs in fragile sites until now. Therefore, we analyzed the enrichments of miRNAs with multiple SNPs in fragile sites of the genome comprehensively and systematically.

**Figure 3:**
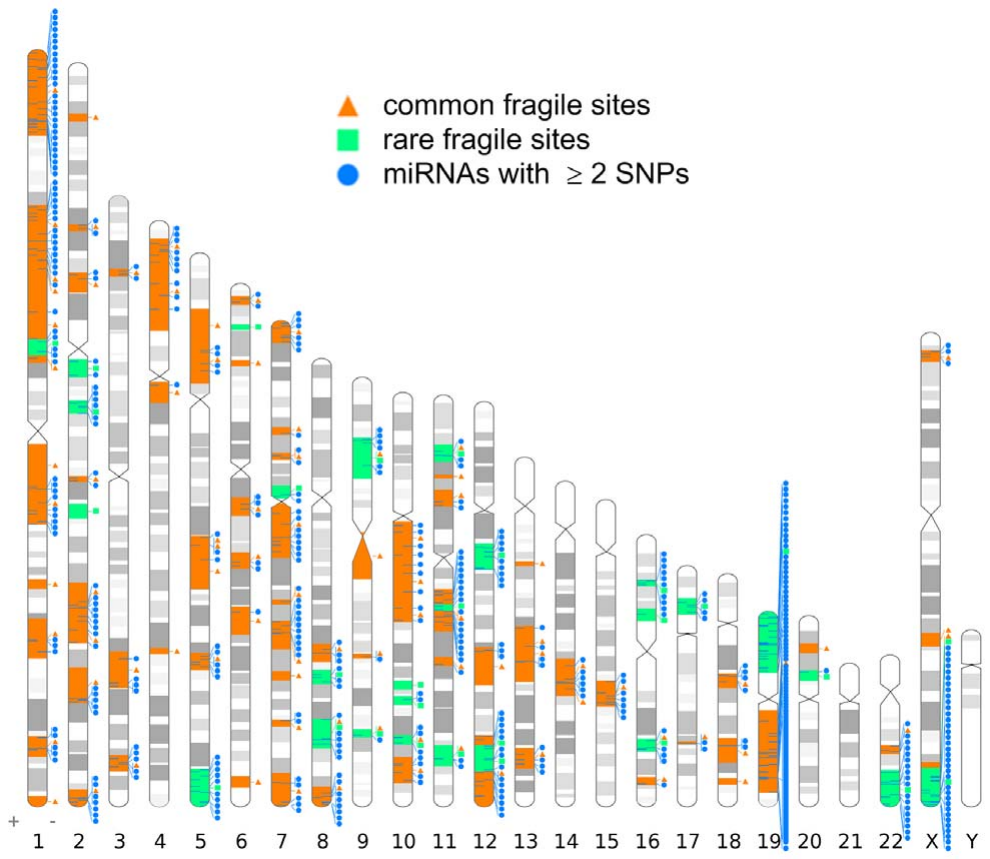
Genome locations of fragile sites and miRNA genes with at least 2 SNPs in them. The karyotype shows the position of 116 fragile sites and 142 miRNAs with at least 2 SNPs in these common fragile sites. The figure is prepared with Idiographica [65].

Based on the latest NCBI Gene database, there are 116 fragile sites in human genome in total, of which 87 are common ones. After comparing the genomic loci of all the human miRNAs to those of fragile sites, 186 of the 510 miRNAs with at least 2 SNPs are located in all fragile sites (Figure 3 and Table S8), which indicates that miRNAs with multiple SNPs are significantly enriched in all fragile sites (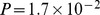, hypergeometric test). The median of SNP densities of miRNAs in all fragile sites is only marginally higher than that of miRNAs out of fragile sites (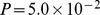, one tailed Mann-Whitney test).

Common fragile sites are present in all individuals, whereas rare fragile sites are present in a small portion of the population with a maximal frequency of 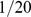 [45]. Notably, 142 out of 186 miRNAs with at least 2 SNPs in all fragile sites locate in common fragile sites, which indicates an even more intensive enrichment of miRNAs with multiple SNPs in common fragile sites (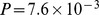, hypergeometric test). Furthermore, the median of SNP densities of miRNAs in common fragile sites is also significantly higher than that of miRNAs out of common fragile sites (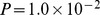, one tailed Mann-Whitney test). Especially, a common fragile site at 19q13, FRA19A, induced by 5-azacytidine, accommodates the largest number of miRNAs with multiple SNPs. Actually, 75 miRNAs locate in this highly conserved fragile site FRA19A in primates [46] and 25 miRNAs of them have at least 2 SNPs. There is another common fragile site, FRA7F in 7q22, which accommodates 10 miRNAs of which six have at least 2 SNPs. On the other hand, miRNAs with more than 1 SNP are not enriched in rare fragile sites (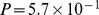, hypergeometric test). Furthermore, the median of SNP densities of miRNAs in rare fragile sites is not significantly higher than that of miRNAs out of rare fragile sites (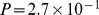, one tailed Mann-Whitney test).

These results indicate that miRNAs with more than 1 SNP are enriched in common fragile sites but not in rare fragile sites. The underlying reasons of this result need further investigations.

### The Effects of SNPs on the Secondary Structures of Pre-miRNAs

The unique hairpin secondary structure is a typical feature of pre-miRNA. SNPs in miRNAs can affect their secondary structures and their functions by reducing or enhancing the expression levels of mature miRNAs [47], [14]. To systematically examine the effects of SNPs on the secondary structures of miRNAs, we calculated the frequencies of 12 different types of substitutions for SNPs in pre-miRNAs, mature miRNAs and seed regions, respectively (see Table 1). We noticed that different types of substitutions have very different frequencies. We tried to explain these differences from three aspects. Firstly, these 12 types of substitutions can be divided into two classes, i.e., transition and transversion. In general, transition is more easily to occur than transversion [48], this could be one important reason of why G 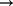 A, C 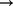 U, A 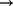 G, and U 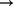 C have higher frequencies than other types of substitutions, as shown in Table 1. Secondly, two kinds of substitutions, A 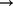 G and C
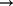
U, are over-represented which might be related to their introductions of G:U Wobble pairs in the secondary structures of pre-miRNAs [49]. Thirdly, U 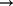 C and G 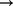 A also have high frequencies, which is probably due to the fact that they might change the original G:U pairs to more stable C-G and A-U pairs, respectively. And these changes could be beneficial to the secondary structures of pre-miRNAs.

**Table 1:** Summary of the number of 12 types of substitutions for SNPs in pre-miRNAs, mature miRNAs and seed regions, respectively.

substitution	seed region	mature miRNA	pre-miRNA
U → G	3	10	44
U → A	2	6	40
G → U	9	19	76
G → C	8	26	78
C → G	6	16	75
C → A	4	12	52
A → U	1	7	34
A → C	4	15	40
subtotal of transversion	37	111	439
U → C	9	44	204
C → U	29	93	355
G → A	35	119	336
A → G	23	59	211
subtotal of transition	96	315	1106
total	133	426	1545

Each cell means the number of substitutions of that row in the regions of that column.

In Table 1, it can also be noticed that the numbers of mutations of A/T 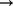 G/C are much smaller than those of G/C 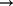 A/T except that A 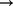 C is a little larger than C 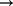 A in mature miRNAs. For example, there are 96 G/C 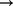 A/T mutations in the seed regions of miRNAs, however there are only 37 A/T 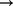 G/C mutations in the same regions. Existing evidences already verified that there tend to be more AT 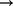 GC mutations in the fast evolving regions [50], [51]. Our results suggest that miRNAs have low evolving speed, presumably due to the functional importance of miRNAs.

**Figure 4:**
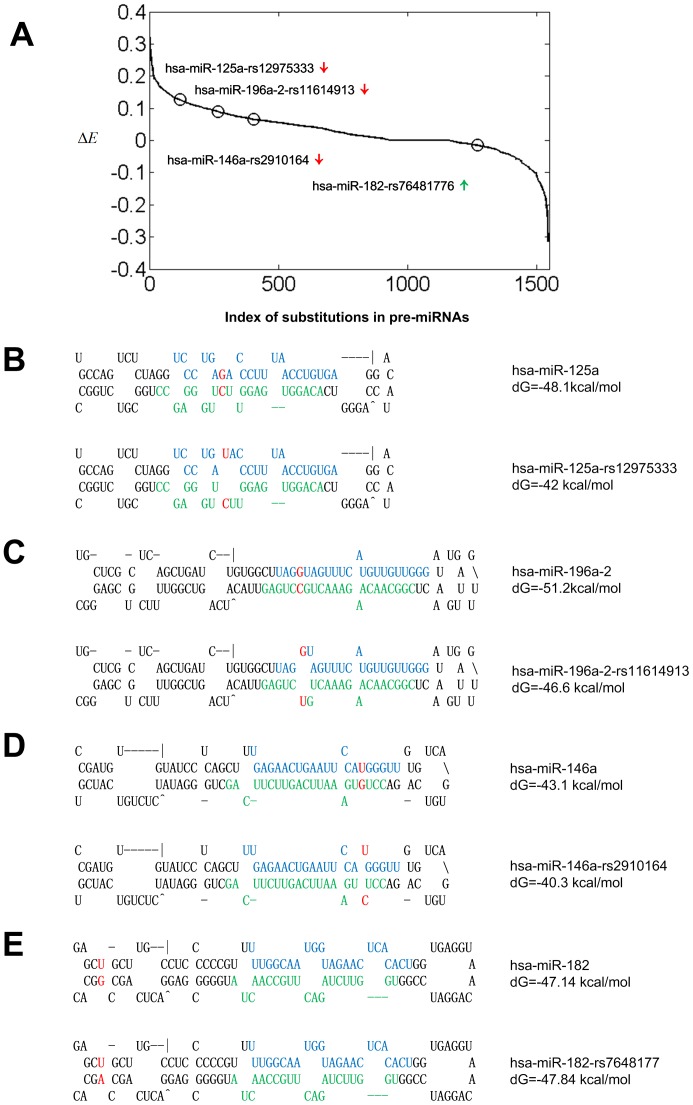
The effects of SNPs on the minimal free energies of secondary structures of pre-miRNAs. Part A shows the distribution of the 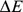 for all the SNPs to the secondary structure of pre-miRNAs. The SNPs here are only substitutions. In part A, the four black circles represent the 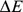 values of four pre-miRNAs with SNPs, i.e., hsa-miR-125a-rs12975333, hsa-miR-196a-2-rs11614913, hsa-miR-146a-rs2910164 and hsa-miR-182-rs76481776 from left to right, respectively. The green and red arrows after the names of miRNAs stand for the up- and down regulations of mature miRNAs in the mutated alleles. There are 1722 substitutions out of 1899 unique SNPs in all 1527 pre-miRNAs. Part B to E show the secondary structure of the four ancestral (upper sections) and mutated pre-miRNAs with SNPs (lower sections) emphasized with circles in part A, respectively. The secondary structures were predicted by Mfold [66]. The regions marked by blue color mean mature miRNAs and the regions marked by green color mean miR*. The bases marked by red color mean SNPs in the pre-miRNAs.

Apart from frequency analysis above, we also evaluated the effects of SNPs on pre-miRNAs by comparing the minimal free energies of of the secondary structures for pre-miRNAs with ancestral alleles and mutated alleles. As has been shown above, some pre-miRNAs may have multiple SNPs. We only considered the effects of substitutions on the stability of pre-miRNAs and evaluated them one by one by assuming that simultaneous appearance of more than one substitution in one miRNA is less likely to happen. As mentioned in Materials and Methods, we treated the alleles in the miRNA sequences downloaded from the miRBase as the ancestral alleles, and the other alleles are considered as mutated alleles. We then calculated the change of minimal free energy normalized to the minimal free energy of ancestral allele, defined as 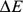 (see Materials and Methods for details), for all the substitutions in pre-miRNAs and the result was shown in Figure 4 and Table S9. Figure 4 shows that some substitutions cause the secondary structure of pre-miRNAs to be unstable, such as rs12975333 in hsa-miR-125a, rs11614913 in hsa-miR-196a-2 and rs2910164 in hsa-miR-146a (see Figure 4B to 4D). In fact, three previous studies have demonstrated that these three substitutions can cause the down-regulations of mature miRNAs [11], [16], [14]. On the other hand, some substitutions make the secondary structures of pre-miRNAs to be more stable, such as rs76481776 in hsa-miR-182 (see Figure 4E). Furthermore, one previous study has demonstrated that this substitution can cause the up-regulation of mature miRNA [47]. Notably, based on our result in Figure 4A, 15% (236/1545) substitutions potentially do not influence the secondary structure in terms of their 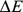.

**Figure 5:**
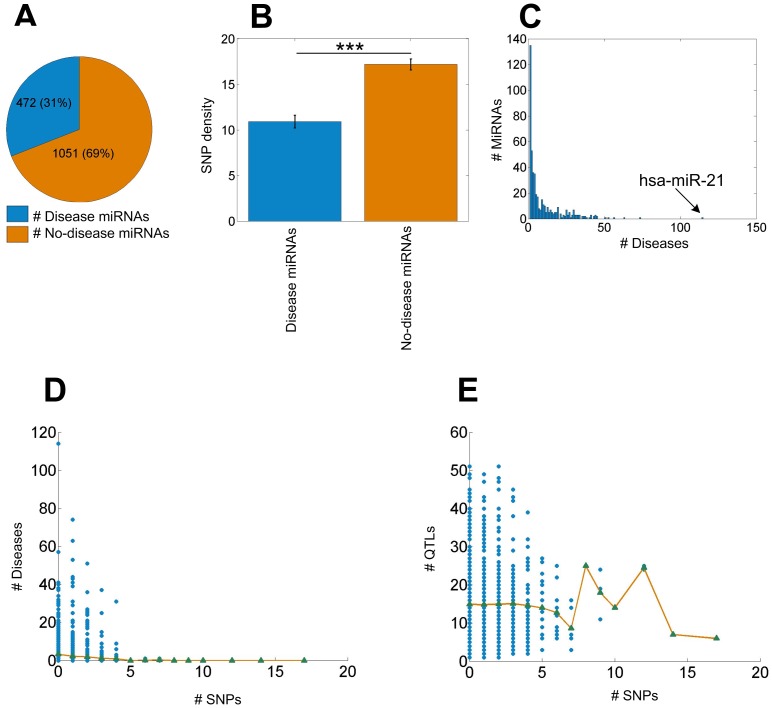
Analysis of SNPs in miRNAs associated with diseases and QTLs. Part A shows the proportion of disease miRNAs in all the miRNAs. Part B shows the comparisons of SNP densities in disease miRNAs and no-disease miRNAs with two sample one tailed 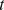 test. In part B, *, ** and *** means 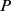-values smaller than 0.05, 0.01 and 0.001, respectively. Error bar indicate the SEM. Part C shows the distribution of the numbers of associated diseases for miRNAs in HMDD. Part D shows the number of SNPs and the number of associated diseases of the miRNAs. MiRNAs are grouped into different groups according to the number of SNPs in them and the average numbers of associated disease for all groups were calculated, shown as green triangles. The green triangles are connected with yellow lines. Part E shows the number of SNPs in the miRNAs and the number of QTLs which the miRNAs are overlapped with. MiRNAs are grouped into different groups according to the number of SNPs in them and the average number of QTLs for each group was calculated, shown as green triangles. The green triangles are connected with yellow line.

Gong *et al.*, [22] recently analyzed the minimal free energies of 785 miRNAs with SNPs. In comparison, we introduced 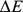 in Equation 2 to clarify that different SNPs may have different effects on the minimal free energies of miRNAs, as shown in Figure 5A. In addition, we also categorized different SNPs based on their nucleotide changes, as shown in Table 1. Finally, the number of miRNAs with SNPs analyzed here are much larger than existing studies [22].

### The Number of SNPs and the Number of Associated Diseases of miRNAs are Negatively Correlated

The HMDD is a database of manually collected relations of miRNAs to diseases, which includes validated de-regulated miRNAs in diseases, miRNAs targeting important oncogenes or tumor suppressors, as well as de-regulated miRNAs found through expression profiles. There are 472 miRNAs, 351 diseases and 4489 miRNA-disease associations in the latest HMDD database (see Figure 5A). In the current study, miRNAs associated with at least one disease in HMDD are considered as disease miRNAs, and miRNAs that are not associated with disease in HMDD are considered as no-disease miRNAs. Figure 5B shows that the average SNP density of disease miRNAs is significantly lower than that of no-disease miRNAs (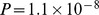, one tailed 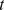-test). Furthermore, Figure 5C shows that the number of associated diseases for each miRNA ranges from 1 to 114. Therefore, we investigated the relationship between the number of SNPs and the number of associated diseases of miRNAs (see Figure 5D and Table S10). There is a significant negative correlation between them (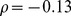, 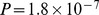, Spearman’s rank correlation test). For example, hsa-miR-21, as a key regulator of oncogenic processes, has been reported be associated with 114 diseases, such as breast cancer, brain cancer, glioblastoma and so on [52], [53], [54], [55], no SNP is found in hsa-miR-21. Another remarkable example is the let-7 family which is associated with numerous diseases [56], [57] and amazingly there is no SNP in all members of the let-7 family in human according to our results. From another direction, there are 51 miRNAs with more than 4 SNPs, but only 2 of them (hsa-miR-1303 [58] and hsa-miR-1234 [59]) have been reported to be associated with diseases. The number of diseases that a miRNA is involved in could be a kind of reflection of its functional importance. Our results again suggest that miRNAs frequently associated with diseases tend to have less SNPs. Furthermore, miRNAs are grouped into different groups according to the number of SNPs in them and the average numbers of associated disease for all groups were calculated, shown as green triangles in Figure 5D. The downward trend of the yellow line is consistent with the negative correlation between the number of SNPs and the number of associated diseases of miRNAs. However, it should be noticed that reports of an association between a miRNA and disease are not independent and are expected to increase after the first report since studies of that miRNA will be motivated by existing results. Therefore, there are some biases in this analysis.

A previous study found that miRNAs with validated seed SNPs overlapped with 830 QTLs in human genome [24]. These results support previous observations that miRNA is an important player in generating genetic variabilities and important genomic sites in the trait’s genetic architecture [24]. There are 1911 QTL regions about 39 different quantitative traits such as blood pressure, body weight, glucose level and so on in the RGD database. Therefore, we examined the relationship between the number of QTLs overlapped with a miRNA and the number of SNPs in it (Figure 5E and Table S11). Interestingly, every miRNA is covered by at least one known QTL region. However, there is no statistically significant correlation between the number of QTLs overlapped with a miRNA and the number of SNPs in it (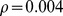, 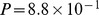, Spearman’s rank correlation test). MiRNAs are grouped into different groups according to the number of SNPs in them and the average number of QTLs for each group was calculated, shown as green triangles in 5E. The fluctuating trend of the yellow line is consistent with the insignificant correlation between the number of QTLs overlapped with a miRNA and the number of SNPs in it. The above result may owe largely to the fact that most QTL regions involve phenotype variation such as body weight but do not cause diseases and thereby do not influence the fitness in most time. Therefore, our results suggest that there are probably no additional natural selection stress on these miRNAs overlapped with more QTL regions.

### Analyzing Frequencies of SNPs in miRNAs Using the Data of the HapMap and 1000 Genome Project

Population differences have been observed in many human complex traits, including disease susceptibility, drug sensitivity and gene expression [60]. Genetic polymorphisms have undergone extensive evaluations for their potential roles in these observed population differences [61], [62], [63]. However, limited endeavor has been done on non-coding RNAs such as miRNAs probably due to a paucity of data for them. One previous paper demonstrated that miRNA expression levels exhibit population differences [60]. As shown previously, the SNPs in miRNAs can affect the stability of pre-miRNAs and then influence the expression of mature miRNAs [12]. Therefore, we hypothesized that the SNPs with significantly different frequencies between different populations may contribute to the observed population difference in miRNA expression to some degree. For this purpose, we identified the SNPs with significantly different frequencies between different populations based on both the HapMap and the 1000 Genome Project data.

**Figure 6:**
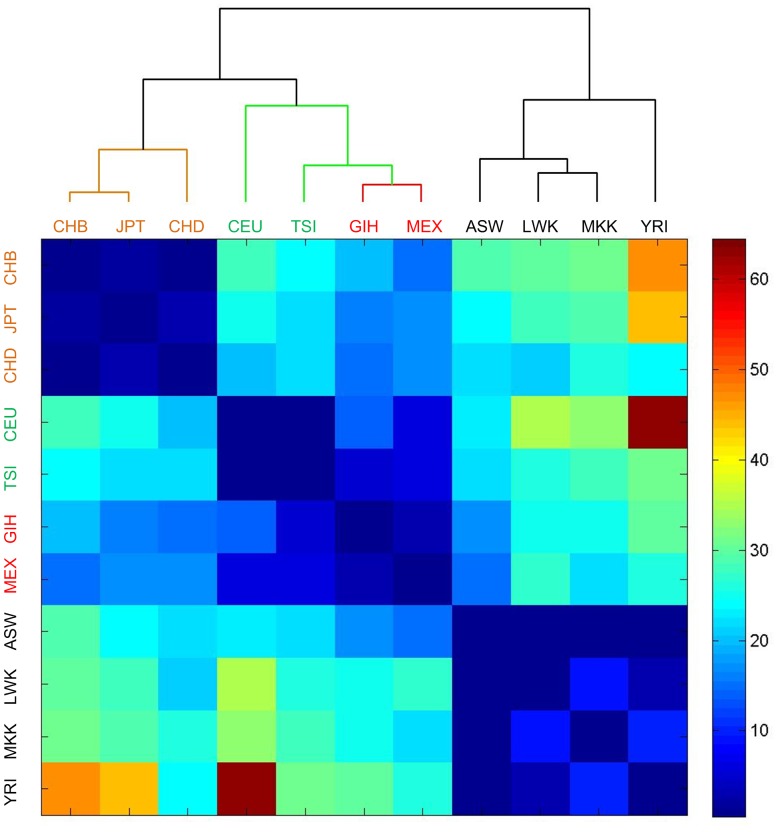
The heat map of the numbers of SNPs in miRNAs with significantly different frequencies between different populations in the HapMap data. The number in a cell means the number of SNPs with significantly different frequencies (with multiple test corrected 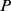-values of smaller than 0.01, see Materials and Methods for details) between the two populations of the row and column. There are 11 populations in the HapMap data. ASW, CEU, CHB, CHD, GIH, JPT, LWK, MEX, MKK, TSI, and YRI stand for African ancestry in Southwest USA; Utah residents (CEPH) with Northern and Western European ancestry; Han Chinese in Beijing, China; Chinese in Metropolitan Denver, Colorado; Gujarati Indians in Houston, Texas; Japanese in Tokyo, Japan; Luhya in Webuye, Kenya; Mexican ancestry in Los Angeles, California; Maasai in Kinyawa, Kenya; Toscani in Italia; and Yoruba in Ibadan, Nigeria, respectively. Among the 11 populations, ASW, LWK, MKK and YRI belong to Africa, marked by blue color; CHB, CHD and JPT belong to Asian, marked by yellow color; CEU and TSI belong to European, marked by green color and GIH and MEX belong to America, marked by red color. The dendrogram was generated with the hierarchical clustering implemented in Matlab.

There are 121 SNPs in pre-miRNAs that have frequency information for at least 2 of the 11 populations in the HapMap database (Table S12). There are 627 SNPs in pre-miRNAs have frequency information for at least 2 of the 4 populations in the 1000 Genome Project database (Table S13). A previous study also collected the frequency information of SNPs in pre-miRNAs, however they only identified 41 SNPs and just presented the frequencies of them without detailed analysis [18]. Here, we identified the SNPs with significantly different frequencies between various populations in the HapMap and 1000 Genome Project (with multiple test corrected 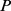-values 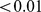) (see Figure 6/Table S14 and Table 2, respectively). From the diagonal of Figure 6, it can be seen that the populations from the same continents have much smaller numbers of SNPs with significantly different frequencies than populations of different continents. Another interesting point lies in that the American and European populations also have very small number of SNPs with significantly different frequencies. Actually, the two American populations are Gujarati Indians in Houston, Texas and Mexican ancestry in Los Angeles, California, respectively. Our results suggest that Gujarati Indians are similar to European populations. And the close relation between European populations and Mexican ancestry is consistent with the migration history of European populations to America. The largest number of SNPs with significantly different frequencies exists between African populations and some European and Asian populations. And the numbers of SNPs with significantly different frequencies between Asian and European (as well as American) populations are not as large as their intersections between African populations. This is probably due to the fact that Asian and European populations are actually living in the same continent. We also performed a hierarchical clustering of populations using their numbers of SNPs with significantly different frequencies between other populations. The obtained dendrogram in Figure 6 suggests that American and European populations have closer relations than other populations; and that the relations between Asian and European/American populations are closer than their relation to African populations. Furthermore, we randomly chose 121 SNPs that have frequency information in at least 2 of the 11 populations in the HapMap data for three times. Then, we also calculated the numbers of SNPs that have significantly different frequencies between different populations, and clustered the 11 populations based on the numbers of these SNPs. The obtained relationships between different populations based on the average number of SNPs with significantly different frequencies between various populations of these three replications are not consistent with their geographical distributions (see Figure S2). These results suggest that SNPs in miRNAs are more likely to be differentiated across populations than a random subset of SNPs of the same size.

**Table 2:** Summary of the number of SNPs with significantly different frequencies between two populations based on the 1000 Genome Project.

	ASN	EUR	AMR	AFR
**ASN**	0	189	166	209
**EUR**	189	0	90	243
**AMR**	166	90	0	294
**AFR**	209	243	294	0

The number in a cell means the number of SNPs with significantly different frequencies (with multiple test corrected 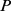-values 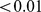, see Materials and Methods for details) between the two populations of the row and column. There are 4 populations in the 1000 Genome Project data. ASN includes the CHB, CHS and JPT; AMR includes CLM, MXL (the same as MEX in the HapMap data) and PUR; AFR includes ASW and LWK; and EUR includes CEU, FIN, GBR, IBS and TSI, respectively. Among these populations, the ASW, CEU, CHB, JPT, LWK, MEX, TSI and YRI represent the same populations in Figure 6. Beside these, CHS, CLM, FIN, GBR, IBS, and PUR stand for Han Chinese South; Colombian in Medellin, Colombia; Finnish from Finland; British from England and Scotland; Iberian populations in Spain; and Puerto Rican in Puerto Rico, respectively.

Similar to those results in Figure 6, Table 2 shows that there are large number of SNPs with significantly different frequencies between AFR and other populations, and also relatively small number of SNPs with significantly different frequencies between AMR and EUR populations. As shown in Table 2, the AMR population includes Colombian in Medellin, Colombia; Puerto Rican in Puerto Rico; and Mexican ancestry in Los Angeles, California. Our results again suggest a close relation between the AMR and EUR populations, presumably due to the migration history of European populations to America. The ASN population and EUR/AMR populations share smaller numbers of SNPs with significantly different frequencies than between the ASN, EUR, AMR and AFR population.

In summary, these results show that the number of SNPs in miRNAs with significantly different frequencies between various populations are consistent with the geographical distributions of these populations. To analyze the history of human population, exiting studies mainly focused the studies of SNPs in protein coding genes. However, our results show that the SNPs in non-coding RNAs, such as miRNAs, could also shed light on our understanding of the evolution of human populations. A previous study has characterized the relationships among the populations by analyzing all autosomal SNPs in genotype data of 988 unrelated individuals with the principal components analysis (PCA) [64]. Our results in Figure 6 are consistent with the results of this study [64], suggesting that SNPs in miRNA genes are a representative set of SNPs that carry the essential information of the relations between different populations. The essential information carried by the SNPs in miRNAs is also supported by the disrupted relationships among the populations inferred from randomly chosen SNPs (see Figure S2).

## Conclusions

In this paper, we performed a genome-wide scan for SNPs in human miRNAs and identified 1899 SNPs in 961 out of 1527 reported pre-miRNAs. To elucidate the distribution of SNPs in human miRNAs, we carefully examined the SNPs in them from several dimensions. Specifically, our results show that (1) conserved miRNAs tend to have lower average SNP densities; (2) clustered miRNAs tend to have lower SNP densities than individual ones; (3) miRNAs with at least two SNPs are enriched in fragile sites; (4) different substitutions of the SNPs in miRNAs have different frequencies which is attributed to their contributions to the stabilities of the secondary structure of pre-miRNAs; and (5) miRNAs frequently associated with diseases tend to have less SNPs. We also found that the average SNP density of miRNAs in intragenic regions is slightly higher than that of intergenic regions, although marginally not statistically significant (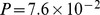, one tailed 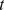-test). These results suggest that conservation, genomic context, secondary structure, and functional importance of human miRNAs affect the accumulations of SNPs in them.

At last, our results also show that the number of SNPs in miRNAs that have significantly different frequencies among various populations in the HapMap and 1000 Genome Project data are consistent with the geographical distributions and migration of these populations. These analyses could provide comprehensive and systematical insights about the distribution of SNPs in human miRNAs and shed light on our understanding of the evolution of human population.

There are some limitations in current study. First, the type of SNPs are not differentiated. In dbSNP, some SNPs have not been verified seriously yet. Thus, a few of these un-verified SNPs might be located in miRNA genes and should be examined before further studies of these SNPs. Second, the conservation of a miRNA is evaluated with the number of species that have this miRNA family. We do this compromise because the incomplete annotation of miRNAs in different species. With better annotation of miRNAs in more species, phylogenetic analysis could be a better method to evaluate the conservation levels of miRNAs.

## Supporting Information

Figure S1 **The distribution of the lengths of human pre-miRNAs.** The lengths of 1527 pre-miRNAs in the miRbase (Release 18) were used. X axis means the length of pre-miRNA (nucleotide), and Y axis means the number of pre-miRNAs.
(TIF)

Figure S2 **The heat map of the numbers of randomly chosen SNPs with significantly different frequencies between different populations in the HapMap data.** The number in a cell means the number of SNPs with significantly different frequencies between the two populations of the row and column. There are 11 populations in the HapMap data. ASW, CEU, CHB, CHD, GIH, JPT, LWK, MEX, MKK, TSI, and YRI stand for African ancestry in Southwest USA; Utah residents (CEPH) with Northern and Western European ancestry; Han Chinese in Beijing, China; Chinese in Metropolitan Denver, Colorado; Gujarati Indians in Houston, Texas; Japanese in Tokyo, Japan; Luhya in Webuye, Kenya; Mexican ancestry in Los Angeles, California; Maasai in Kinyawa, Kenya; Toscani in Italia; and Yoruba in Ibadan, Nigeria, respectively. Among the 11 populations, ASW, LWK, MKK and YRI belong to Africa, marked by blue color; CHB, CHD and JPT belong to Asian, marked by yellow color; CEU and TSI belong to European, marked by green color and GIH and MEX belong to America, marked by red color. The dendrogram was generated with the hierarchical clustering implemented in Matlab.
(TIF)

Table S1 **Summary of SNPs in pre-miRNAs.** The information of the columns is given in the second sheet.
(XLS)

Table S2 **Summary of SNPs in mature miRNAs.** The information of the columns is given in the second sheet.
(XLS)

Table S3 **Summary of SNPs in seed regions of miRNAs.** The information of the columns is given in the second sheet.
(XLS)

Table S4 **Conservation analysis of pre-miRNAs.** The information of the columns is given in the second sheet.
(XLS)

Table S5 **Conservation analysis mature miRNAs.** The information of the columns is given in the second sheet.
(XLS)

Table S6 **Conservation analysis seed regions.** The information of the columns is given in the second sheet.
(XLS)

Table S7 **Summary clustered miRNAs and individual miRNAs.** This sheet lists the clustered miRNAs, and individual miRNAs are listed in the second sheet. The information of the columns is given in the third sheet.
(XLS)

Table S8 **Summary of SNPs in or not in fragile sites.** The information of the columns is given in the second sheet.
(XLS)

Table S9 **The minimal free energy of pre-miRNAs with reference and mutated alleles.** The information of the columns is given in the second sheet.
(XLS)

Table S10 **Summary the number of diseases associated with the pre-miRNAs.** The information of the columns is given in the second sheet.
(XLS)

Table S11 **Summary the number of QTLs overlapped with the pre-miRNAs.** The information of the columns is given in the second sheet.
(XLS)

Table S12
**Summary the 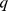 values of the SNPs between two populations based on the HapMap data.** The information of the columns is given in the second sheet.(XLS)

Table S13Summary the 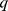 values of the SNPs between two populations based on the 1000 Genome Project data. The information of the columns is given in the second sheet.(XLS)

Table S14 **Summary the number of SNPs with significantly different frequencies between two populations based on the HapMap data.** The information of the columns is given in the second sheet.
(XLS)
