# Supplementary figures and images for: Comprehensive Analysis of Single Nucleotide Polymorphisms in Human MicroRNAs
Source: PLoS One. 2013 Nov 5;8(11):e78028. doi: 10.1371/journal.pone.0078028 (PMC3818353; doi:10.1371/journal.pone.0078028)

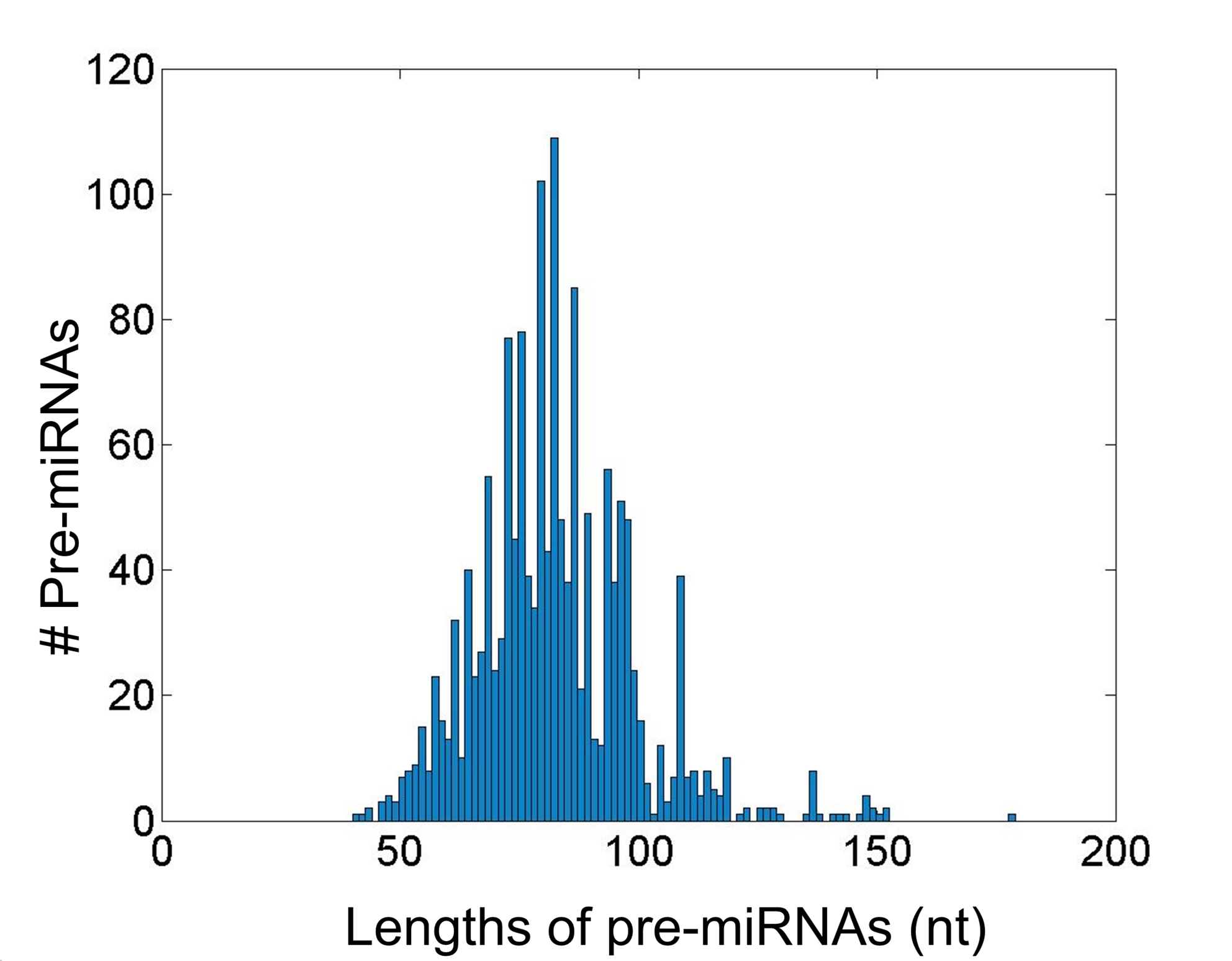

Supplement: Figure S1 — The distribution of the lengths of human pre-miRNAs. The lengths of 1527 pre-miRNAs in the miRbase (Release 18) were used. X axis means the length of pre-miRNA (nucleotide), and Y axis means the number of pre-miRNAs. (TIF) [file pone.0078028.s001.tif]

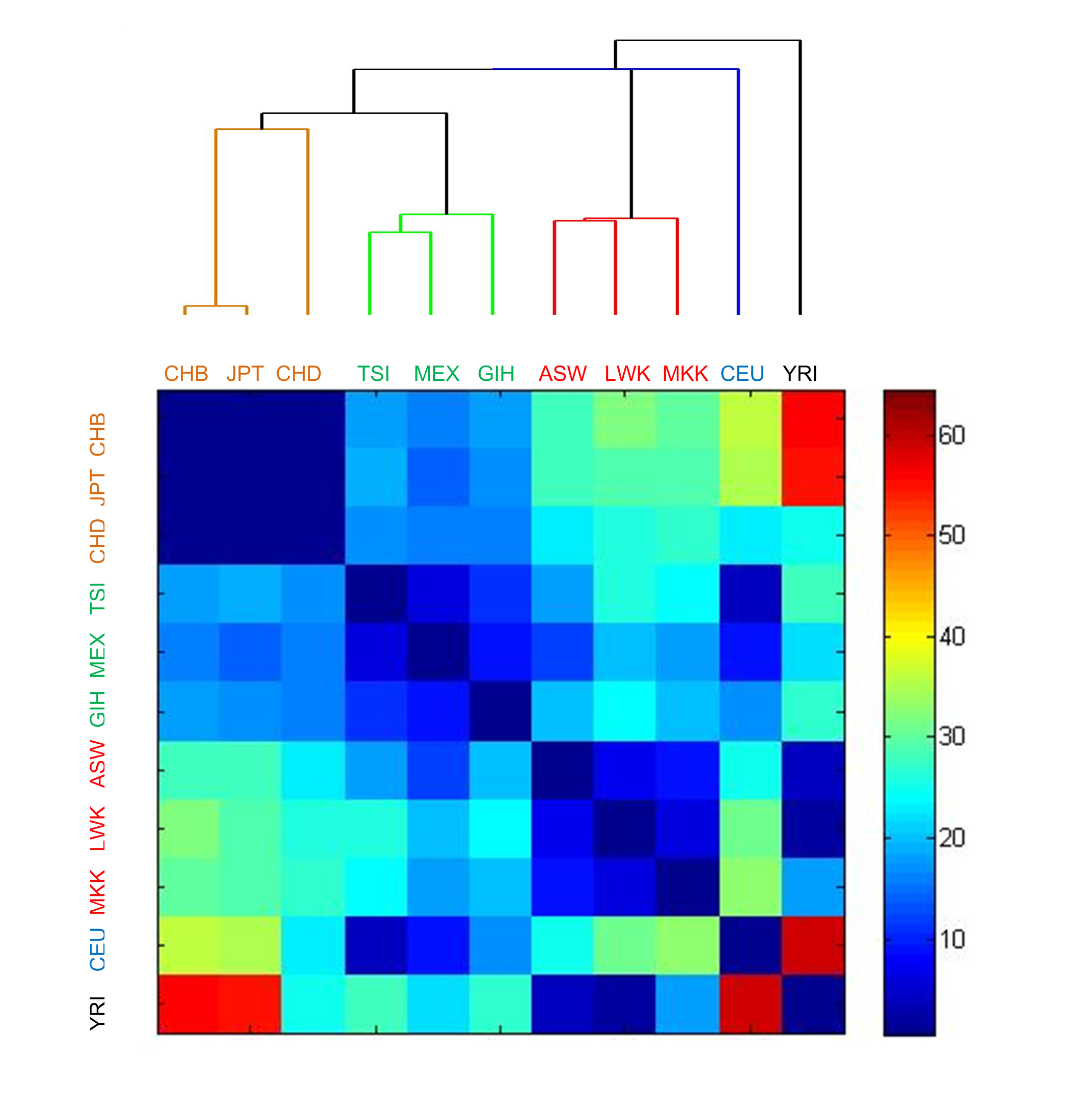

Supplement: Figure S2 — The heat map of the numbers of randomly chosen SNPs with significantly different frequencies between different populations in the HapMap data. The number in a cell means the number of SNPs with significantly different frequencies between the two populations of the row and column. There are 11 populations in the HapMap data. ASW, CEU, CHB, CHD, GIH, JPT, LWK, MEX, MKK, TSI, and YRI stand for African ancestry in Southwest USA; Utah residents (CEPH) with Northern and Western European ancestry; Han Chinese in Beijing, China; Chinese in Metropolitan Denver, Colorado; Gujarati Indians in Houston, Texas; Japanese in Tokyo, Japan; Luhya in Webuye, Kenya; Mexican ancestry in Los Angeles, California; Maasai in Kinyawa, Kenya; Toscani in Italia; and Yoruba in Ibadan, Nigeria, respectively. Among the 11 populations, ASW, LWK, MKK and YRI belong to Africa, marked by blue color; CHB, CHD and JPT belong to Asian, marked by yellow color; CEU and TSI belong to European, marked by green color and GIH and MEX belong to America, marked by red color. The dendrogram was generated with the hierarchical clustering implemented in Matlab. (TIF) [file pone.0078028.s002.tif]
